# The Uses and Limitations of Surgery

**Published:** 1905-04-15

**Authors:** 


### The Hospital.

A Journal of

The Medical Sciences and Hospital Administration.

Vol. XXXVIII.—No. 968. SATURDAY, APRIL 15, 1905.

### The Uses and Limitations of Surgery.

We place before our readers to-day two papers on the uses and limitations of surgery, respectively by Dr. Essex Wynter and Mr. D'Arcy Power; and which, therefore, approach one of the most difficult questions which can offer itself to the practitioner from the several points of view of the physician and of the surgeon. The obituary notices of the daily papers have for some time past contained the statement "after an operation" with what is regarded by the majority of non-medical readers as quite an undue degree of frequency, and it would be idle to deny that, even in the profession itself, there has been some amount of fear lest the resources of surgery were being applied with undue frequency, or to conditions in which failure might have been confidently foretold by experience. An "operation" which is not justified by the event constitutes a very formidable addition to the anxieties which illness inflicts upon the families in which it occurs; and every medical attendant must now and then be placed in a position of serious responsibility with reference to the time and the occasion for which surgical assistance is imperatively demanded. The difficulty chiefly arises, at the present day, with reference to abdominal conditions; in which, on the one hand, it is obviously possible either to lose time which cannot be recalled, or, on the other, to hasten operative proceedings which might have been omitted, and which, even if they do not precipitate a fatal event, may yet leave behind them a number of troublesome and embarrassing complications. In these circumstances, it is but natural that a surgeon should address himself chiefly to the conditions which determine the necessity for operating, and the physician to those which render it justifiable to refrain; and it must remain for the reader to bring their respective precepts into harmony, and to apply them to the ever varying symptoms by which he will inevitably be confronted in the sick room.

It is, of course, of the essence of the question, as regards abdominal surgery, that its successes and its failures are alike of recent date. If this fact be borne constantly in mind, the results of abdominal diseases as they were witnessed during the time prior to the introduction of asepticism may perhaps be regarded as a dominant factor in the discussion. We believe it to be true that they were followed by a much larger percentage of cures or of recoveries than modern practitioners always realise, and that the perityphlitis or peritonitis of the second quarter of the last century frequently afforded very remarkable examples of the power of Nature to assert herself under favourable conditions. Such a book as Watson's "Lectures on the Practice of Physic" contains narratives of recoveries from forms of disease which now, if they could be accurately diagnosed, would be regarded either as desperate or as admitting of relief only by operative methods; and hence are calculated to afford much encouragement to the physician who decides to wait. It is at least equally certain that the post-mortem table too frequently displays lesions with which surgery might have reasonably hoped to deal successfully, and hence that lives have presumably been lost for want of the aid which it was calculated to afford. It is between this Scylla and this Charybdis that the practitioner of to-day constantly finds himself called upon to steer; and the papers to which we refer, and which are written by pilots who have sounded every portion of the channels with which they deal, can hardly fail to afford valuable help in arriving at a decision upon which the whole future of the patient or of his family must so frequently depend.

It is equally insisted upon by both writers and may, perhaps, be regarded as almost self-evident, that this decision must really turn upon two factors: first, upon an accurate diagnosis of the physical condition to be remedied, and, secondly, upon a correct estimation of the vital powers of the sufferer. It is obviously useless to have resort to mechanical means for a state of things which is not mechanically remediable, and it is obviously useless to perform a mechanically successful operation upon a patient who will not survive the shock of its performance or the injury inflicted by the diseased condition which it is designed to rectify. Dr. Wynter and Mr. Power alike contribute of their experience in order to indicate the respective values of various symptoms. We doubt very much, however, whether the most observant and most experienced practitioner would always find it easy, or even possible, to classify the symptoms of a given case in the order of their values, or to assign the precise reasons which might lead him even to a decided conclusion. There is a certain skill in the appreciation of the state of a patient which comes only from long and careful observation, and the bases of which must often be incommunicable, but for the highest degree of which certain natural gifts appear to be indispensable. Practitioners of experience are not slow to recognise the cultivated clinical insight which seems to have its foundation in some instinctive power of perception, trained to the most complete possible development, and which, to mention only those who are no more with us, was so conspicuously displayed by Jenner and by Paget. It is by an earnest endeavour to follow their examples that the best security will be afforded against the errors of haste on the one hand or the errors of delay on the other. It should never be absent from the mind of the surgeon that he incurs a responsibility, in operating, which only the most complete endeavour to grasp the whole of the conditions will render it right for him to accept; and it should never be absent from the mind of the physician that his reliance upon the reparative processes of nature may be excessive or misplaced. It is at least permissible to hope that, in both, the power of avoiding error will be steadily increased by the conscientious utilisation of experience.

